# Evaluation of User Interfaces for Actuated Control of Endoscopes During Flexible Endoscopy

**DOI:** 10.1109/JTEHM.2026.3659651

**Published:** 2026-01-30

**Authors:** Sofia Basha, Mohammad Khorasani, Nihal Abdurahiman, Jhasketan Padhan, Victor Baez, Abdulla Al-Ansari, Panagiotis Tsiamyrtzis, Aaron T. Becker, Nikhil V. Navkar

**Affiliations:** Department of SurgeryHamad Medical Corporation Doha Qatar; Electrical and Computer EngineeringUniversity of Houston 14743 Houston TX 77204 USA; Department of Mechanical EngineeringPolitecnico di Milano Milan 20133 Italy; Department of StatisticsAthens University of Economics and Business 59164 Athens 10434 Greece

**Keywords:** Human–computer interface, flexible endoscopy, colonoscopy

## Abstract

Objective: Flexible endoscopy is a valuable tool in diagnostic procedures, enabling examination of internal areas via natural orifices. An actuation system tends to improve the procedural outcomes by enabling controlled movements of the endoscope and offering a stable view of the operative field. A user interface is used to issue actuation commands to these systems. Thus, selection of an ideal user interface is vital to improve the ergonomics for the endoscopist and to ensure efficient endoscope navigation. The objective of this work is to perform an in-depth comparative analysis of various user interfaces to optimize endoscope maneuverability. Methods and Procedures: A custom-built actuation system was used to maneuver a flexible endoscope. The actuation system enabled translational and rotational movement of the endoscope’s shaft as well as supported left/right and up/down steering of the endoscope’s distal end. Four user interfaces (head-motion based device, eye-gaze based device, a stylus, and a joystick) working under three interaction modes (continuous, discrete, threshold) along with a clutching mechanism were used to issue commands to the actuation system. A user study was conducted to assess the effectiveness of the user interfaces for two scenarios: Scenario-A, which involved maneuvering the endoscope’s distal end to focus on a localized operative field, and Scenario-B, which required targeting polyps during the withdrawal phase of a simulated colonoscopy. Results: In Scenario-A, the head motion-based device and stylus, when used in continuous interaction mode, resulted in the shorter task duration and fewer clutches. The joystick, operating under threshold interaction mode, also demonstrated a reduced task duration. Additionally, the joystick led to fewer instances of the endoscope’s focus shifting outside the localized operative field. In Scenario-B, eye gaze-based device under discrete interaction mode took the longest duration for task completion. The continuous mode of the stylus took the shortest duration to target polyps, once visualized in the operating field. However, it also required the highest number of clutches compared to other user interface and interaction modes. Conclusion: The joystick consistently outperformed other interfaces across all interaction modes. Performance among the other user interfaces varied based on the parameters of the scenarios. Head motion-based and eye-based user interfaces enabled hands-free manipulation of the endoscope. This study establishes a benchmark for enhancing both user interfaces and interaction modes in actuated flexible endoscopy.

Clinical and Translational Impact:—The user study presents a detailed comparison to assess the effectiveness of different user interfaces of an actuation system for manipulating endoscopes during flexible endoscopy. The outcomes of this study can drive advancements in actuated endoscopic technologies, particularly from an ergonomic standpoint, thereby enhancing both diagnostic and therapeutic procedures.

## I. Introduction

Flexible endoscopy has been used in the healthcare field for a considerable amount of time [1]. In present day, its usage is not only limited to visualization for diagnosis but has also been extended to therapeutic procedures, encompassing both transluminal and endoluminal techniques through the natural orifices of the human body [2]. The capability to perform these procedures through natural orifices (i.e., mouth, anus, urethra and vagina) and body passages allows greater reach without the need for an incision. The flexible nature reduces tissue trauma and provides better visualization due to the ability to place the camera near the targeted tissue. These advantages have contributed to the clinical success of flexible endoscopy.

Despite these advantages, it is difficult for endoscopists to maneuver flexible endoscopes due to (a) the need to manually support the weight of the endoscope throughout the procedure, (b) the complexity of manipulating the rotating knobs in the control section of the endoscope, and (c) inaccurate transfer of force from the endoscope’s rear end to its distal end. Apart from cognitive workload, experienced endoscopists frequently report musculoskeletal issues in back, neck, shoulders, and hands arising from the repetitive nature of the diagnostic procedures (such as upper GI endoscopy and colonoscopy) [3], [4], [5]. To reduce the cognitive workload and improve the ergonomics for an endoscopist, actuation systems for flexible endoscopy have been proposed [6], [7]. These actuation systems employ a controller-manipulator design. The endoscopist uses the controller console (a) to view the diagnostic field on a visualization screen and (b) to provide actuation commands using a user interface. These actuation commands trigger the actuation system and causes movements of the endoscope within body passages. Therefore, to achieve precise control of an endoscope, a selection of an ideal user interface is necessary. An ideal user interface would facilitate efficient maneuvering and accurate positioning of the endoscope [8]. Working towards this direction, the study presents a comparative analysis that evaluates different user interfaces operating under various interaction modes for maneuvering flexible endoscopes. The proposed system achieves a Technology Readiness Level (TRL) of 6, indicating its demonstration in a relevant clinical environment. At this level, the system is tested as an integrated unit in terms of performance, robustness, and usability under simulated clinical conditions that closely mimic real-world workflows and constraints.

## II. Related Works

A comprehensive literature search was conducted across major academic databases (e.g., IEEE Xplore, PubMed, ScienceDirect, SpringerLink and ACM Digital Library), focusing on peer-reviewed studies involving flexible endoscopes controlled through diverse user interfaces. Table 1 presents an overview of both commercial products and research prototypes of actuation systems designed for flexible endoscopy, all of which remain in active use. These systems facilitate the usage of various types of endoscopes (such as ureteroscope, gastroscope, bronchoscope, colonoscope, and uretero-renoscope) and are used across a range of procedures including flexible ureteroscopy, gastroscopy, and accessing the upper GI tract [15], [17], stomach [18], [21], [22], and colon [19]. While the actuation systems vary in design depending on their intended usage, their manipulation mechanisms are consistent. They provide up to four degrees-of-freedom for endoscope movement and include: (a) left/right steering of the distal end, (b) up/down movement of the distal end, (c) rotational movement of the endoscope’s shaft, and (d) insertion/retraction of the endoscope’s shaft. A user interface allows an endoscopist to provide the necessary actuation commands to enable these endoscope movements.

**TABLE 1:** User Interfaces Used in Existing Commercial Products and Research Prototypes for Actuated Flexible Endoscope Manipulation

	Actuation System	Type of Endoscope	Procedure	Endoscope Movements*				Interface
				L/R	U/D	I/R	Rot	
Product	Endodrive [9]	All types	Endoscopy	-	-	$\checkmark $	-	Foot switch
	Avicenna–[10]	Ureterorenoscope	Flexible Ureteroscopy	-	$\checkmark $	$\checkmark $	$\checkmark $	Joystick
	ILY ® [11]	Flexible Ureteroscope	Flexible Ureteroscopy	-	$\checkmark $	$\checkmark $	-	Joystick
	EasyUretero [12]	Flexible Ureteroscope	Flexible Ureteroscopy	-	$\checkmark $	$\checkmark $	-	Stylus
Research Prototype	RAFE [13]	Gastroscope	ESD (Human)	$\checkmark $	$\checkmark $	$\checkmark $	$\checkmark $	Stylus
	J. Zhao et al. [14]	Flexible Ureteroscope	Flexible Ureteroscopy (Human)	$\checkmark $	$\checkmark $	$\checkmark $	$\checkmark $	Custom built
	R. Reilink et al. [15]	Gastroscope	Gastroscopy (Phantom)	$\checkmark $	$\checkmark $	-	-	Head motion
	Y. X. Mak et al. [16]	Bronchoscope	Generic (Phantom)	-	$\checkmark $	-	$\checkmark $	Head motion
	A. Sivananthan et al. [17]	Gastroscope	Upper GI tract (Phantom)	$\checkmark $	$\checkmark $	$\checkmark $	$\checkmark $	Multiple
	iGaze2 [18]	Gastroscope	Stomach (Phantom)	$\checkmark $	$\checkmark $	-	-	Eye gaze
	R. Reilink et al. [19]	Simulated Endoscope	Colon (Phantom)	$\checkmark $	$\checkmark $	-	-	Stylus
	J. G. Ruiter [20]	Colonoscope	Generic (Phantom)	$\checkmark $	$\checkmark $	$\checkmark $	$\checkmark $	Stylus
	P. Allemann et al. [21]	Gastroscope	Stomach (Anesthetized animals)	$\checkmark $	$\checkmark $	$\checkmark $	$\checkmark $	Joystick
	S. Basha et al. [22]	Gastroscope	Stomach (Phantom)	$\checkmark $	$\checkmark $	$\checkmark $	$\checkmark $	Joystick

^*^ Movements of the endoscope: L/R - Left/Right, U/D - Up/Down, I/R - Insert/Retract, and Rot - Rotate

The first commercial endoscope actuation system, EndoDrive [9], used a foot pedal for insertion and retraction of the shaft and was compatible with all types of endoscopes. However, since it provided control for only one degree-of-freedom, subsequent actuation systems introduced user interfaces that enabled movements across all degrees-of-freedom. These included styluses [12], [13], [19], [20], joysticks [10], [11], [21], [22], head motion based controls [15], [16], and eye-gaze controls [18]. In the actuation system proposed by Sivananthan et al. [17], multiple user interfaces were combined to enable different endoscope movements. This included eye tracking to control the distal end, head motion for endoscope rotation, and joystick for insertion/retraction of the endoscope. A custom built user interface was used by Zhao et al. [14] that mimicked an aircraft yoke (control wheel) enabling translation, rotation and bending of a flexible ureteroscope. In summary, the commercial systems were primarily designed for flexible ureteroscopy, and required only one movement (up/down) of the distal end. In these systems, joysticks and styluses were commonly employed. In contrast, research prototypes of actuation system were designed to accommodate various types of endoscopes and utilized a range of user interfaces.

Apart from the development of actuated systems using individual user interfaces, as presented in Table 1, several previous works have also conducted comparative studies between different user interfaces for remotely manipulating flexible endoscopes. For example, Finocchiaro et al. [23] compared a joystick and a stylus in a virtual colonoscopy task. However, the virtual environment was not able to fully replicate real-world challenges, such as the increased torque required to rotate the knobs when the tip reaches close to the end position, or the support needed for the scope shaft outside the cavity. Other studies [24], [25] compared joystick and touchpad for robotic endoscope control, revealing mixed results for the user interfaces. The studies did not report any statistical difference among both the objective and subjective evaluation of the two interfaces. This could be due to the limited number of parameters evaluated. Another study by Mak et al. [16] examined the performance of manual operation versus a headband-mounted IMU interface along with a HoloLens. This work was limited to the rotation and bending mechanism of a bronchoscope and was evaluated in a non-anatomical spherical phantom. Although these studies focused on automating endoscope motion and compared up to two user interfaces, to the best of our knowledge, no research has simultaneously compared multiple user interfaces. In this study, we present a detailed comparison of different user interfaces employed in remote manipulation of a flexible endoscope in both 2D and 3D space using path following and targeting tasks. In addition, we introduced a new interaction mode, named continuous, apart from the existing discrete and threshold interaction modes commonly used in existing user interfaces (details presented in Table 2).

**TABLE 2:** Interaction Modes

Interaction Modes	Working of the interaction modes
Continuous (C)	The position of operator’s head (or the stylus tip) is mapped to the distal end of the endoscope. As the operator moves his/her head (or the stylus tip), the distal end of the endoscope follows the motion, leading to multidirectional continuous movements.
Discrete (D)	The input provided via the user interface translates into the endoscope’s distal end motion along a discrete direction (i.e., +X, -X, +Y, -Y, +Z, or -Z) with a constant velocity. This enables the operator to provide unidirectional commands (up, down, left, right, in, and out) one at a time.
Threshold (T)	The user interface acts as a three-axis gimbal. If the new position recorded by the user interface exceeds the initial position by a threshold, the distal end of the endoscope starts drifting in that direction at a constant velocity.

## III. Methodology

### A. Actuation System

**FIGURE 1.**
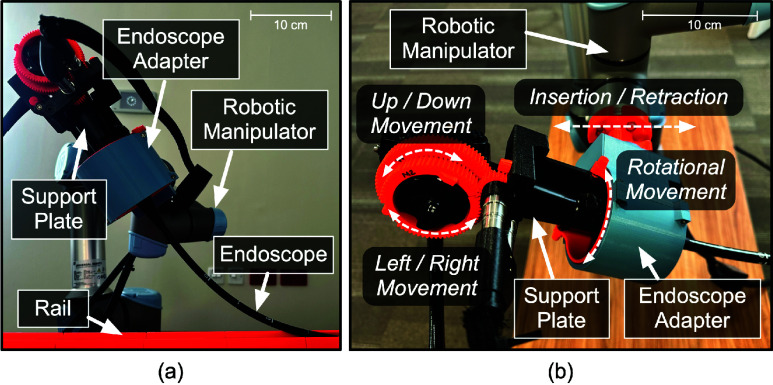
(a) Actuation system used in the study comprising of endoscope adapter, support plate, and robotic manipulator. (b) Manipulation of the endoscope caused by the different components of the actuation system.

**FIGURE 2.**
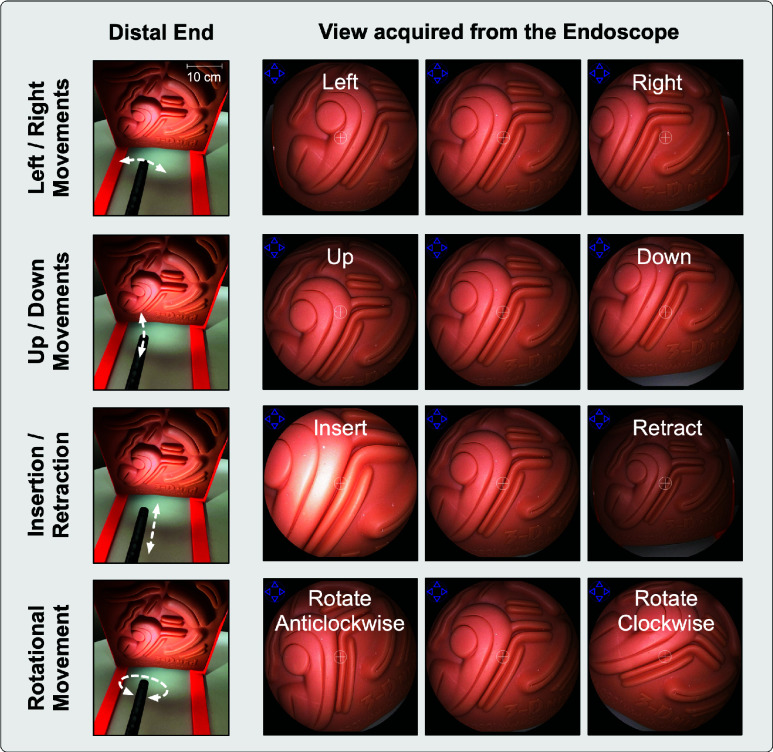
Changes in the view acquired by the endoscope caused by the movements of endoscope’s distal end.

In the proposed study, an actuation system was used to maneuver the endoscopes (Figure 1a). The actuation system was based on our previous design [22], and comprised of three main components: an endoscope adapter [26], a support plate, and an off-the-shelf robotic manipulator (UR5e–Universal Robots). The endoscope was placed on the support plate, which was then inserted into the endoscope adapter attached to a robotic manipulator. The support plate consisted of two motors that engaged with the up/down and left/right rotating knobs via custom 3D printed gears, while the endoscope adapter consisted of one motor that enabled the endoscope rotation. Thus, the overall setup enabled the endoscope’s up/down, left/right, rotational, and insertion/retraction motions without any manual intervention. Each component caused different manipulation of the endoscope (Figure 1b). The support plate enabled up/down and left/right movement of the distal end of the endoscope. The endoscope adapter facilitated the rotational movement of the endoscope along its shaft, whereas the robotic manipulator allowed insertion and retraction of the endoscope (using a rail). These movements actuate the endoscope’s distal end, altering the acquired view (as shown in Figure 2).

### B. User Interfaces

**FIGURE 3.**
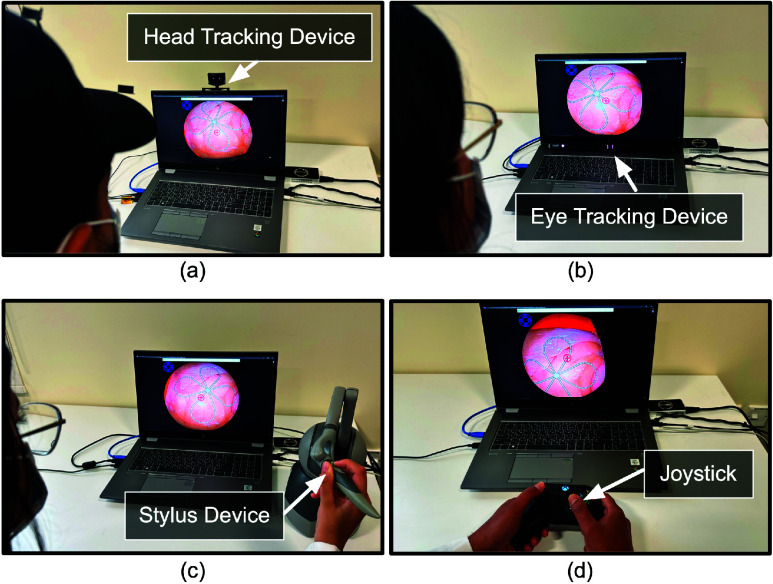
The user interfaces used in the study for control of the endoscope included (a) head tracking device, (b) eye tracking device, (c) stylus device, (d) joystick.

Four commonly used user interfaces (shown in Figure 3) were used to provide commands to the actuation system for maneuvering the endoscope. The user interfaces were based on head motion (using a head tracking device, Track IR–Natural Point), eye gaze (using an eye tracking device, Tobii eye tracker), hand motion (using a stylus device, Touch Haptic–3D Systems), and thumb motion (using a gamepad joystick, Xbox controller–Microsoft). The user interfaces operated under three distinct interaction modes, namely, continuous, discrete and threshold. The working of each interaction mode is presented in Table 2. The mapping of the user interfaces with actuation commands provided by the operator is presented in Table 3. As shown in Table 3, certain interface–interaction mode combinations were omitted due to feasibility and design limitations. Specifically, the Eye Gaze–Continuous and Joystick–Continuous combinations were excluded. In the case of Eye Gaze, continuous movement capture resulted in rapid and often unintentional eye motions, leading to unstable and unpredictable system responses. Furthermore, the precision of the eye-tracking system was insufficient to reliably interpret fine-grained, continuous movements, affecting control accuracy. For the Joystick interface, the limited physical range of motion made it impractical to capture user-controlled speed variations necessary for continuous operation.

**TABLE 3:** User Interfaces

User Interface	Interaction			Clutch	Mapping of user interface to the movements of endoscope
	C	T	D		
Head Motion	$\checkmark $	$\checkmark $	$\checkmark $	Used	Roll, yaw, and pitch of the operator’s head are mapped to rotation of the endoscope, left/right movement, and up/down movement. Translational motion of head is mapped to endoscope’s insertion/retraction.
Eye Gaze		$\checkmark $	$\checkmark $	Used	An infrared optical sensor is used to track the operator’s eye motion. The eye gaze of the operator is mapped to the distal tip angulation of the scope and the head motion is used for shaft rotation and insertion/retraction.
Stylus	$\checkmark $	$\checkmark $	$\checkmark $	Used	Translation of the stylus tip causes left/right movement, up/down movement, and insertion/retraction motion. Rotating the stylus along the shaft causes rotation of the endoscope.
Joystick		$\checkmark $	$\checkmark $	Not Used	Operator uses left and right thumb to move the sticks of the joystick. One stick controlled the left/right and up/down movement, whereas the other stick controlled the insertion/retraction and rotation of the endoscope.

In addition to the user interfaces, a clutch was used with the actuation system. A foot pedal (Savant Elite2–Kinesis) was used as a clutch for head motion and eye gaze-based user interfaces, whereas the button on the stylus device was used as a clutch for the hand motion-based user interface. The clutch allowed the operator to halt the endoscope’s movement, ergonomically reposition themselves as needed, and then re-engage when ready to resume movement of the endoscope. In the case of the joystick, releasing the thumb sticks returned it to a neutral position, halting the movement without the need for an additional clutch.

### C. User Study

The user-study was conducted with 28 subjects (14 males and 14 females, aged between 22 and 40 years) from the Department of Surgery at Hamad Medical Corporation. The study was approved by the institutional review board ethical committee (Medical Research Center, Doha, Qatar, approval number MRC-01-20-087). The participants involved in the study were academic researchers with extensive experience in the development of advanced surgical technologies. Also, the subjects were selected ensuring that they did not have any previous experience with user interfaces, such as gaming experts. The subjects underwent a preparatory session to understand the mapping of the input provided via the user interface with the movement of the endoscope. This session introduced the ten different input methods, and the subjects tested each of them to become familiar with their working mechanisms. The session concluded once the subjects demonstrated confidence in using the controls, typically taking about 30 minutes per subject before beginning the actual study. Two scenarios, Scenario-A and Scenario-B, were designed to evaluate the user interfaces under different interaction modes. The scenarios were fixed throughout the study for all subjects. To ensure that there is no learning effect, a combined randomized between-subjects and within-subjects experimental protocol was used. The subjects were randomly divided into two groups of 14, with each group assigned to one of the scenarios. Within each group, every participant performed the task once for each of the interface–interaction mode combinations. The order of testing was randomized individually for each participant to minimize order effects.

**FIGURE 4.**
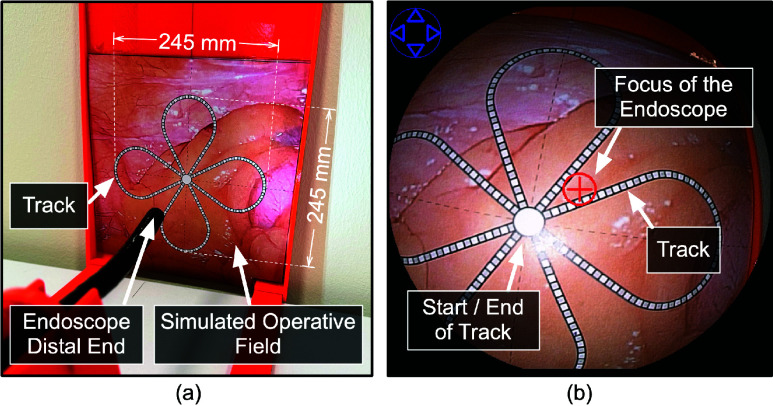
(a) Experimental setup used for Scenario-A. (b) View of the simulated operative field acquired from the endoscope.

Scenario-A assessed the user interfaces and the interaction modes for bending the endoscope’s distal end using up/down and left/right movements. These movements are used to focus on a selected target while keeping the endoscope’s shaft stationary. The experimental setup consisted of a real endoscope connected to the proposed actuation system, with movement limited to the distal end of the scope. The scenario’s task involved following a track printed on an image with a tissue-like background simulating an operative field. This physical image was placed orthogonally in front of the endoscope, and the live camera feed was displayed to the user on a screen. Figure 4a shows the experimental setup with the endoscope facing the printed image, while Figure 4b shows the endoscopic view seen by the user. The track is a four-leaf clover shape that includes segments that mimic the S-shapes, C-shapes, and straight line paths in [27] and [28]. A circled plus sign “ $\oplus $” was rendered at the center of the operative field view to show the focus of the endoscope (Figure 4b). The task involved traversing the track, while ensuring the endoscope focus stays on the track. The parameters recorded for this scenario were: (a) the duration to traverse the path (minutes), (b) the count of how many times endoscope’s focus shifts outside the track, and (c) the clutch count while traversing the track. A mentor was present during the study to ensure that the participant returned to the same segment of the track from where a deviation occurred. The number of such deviations was recorded as the focus shift count.

**FIGURE 5.**
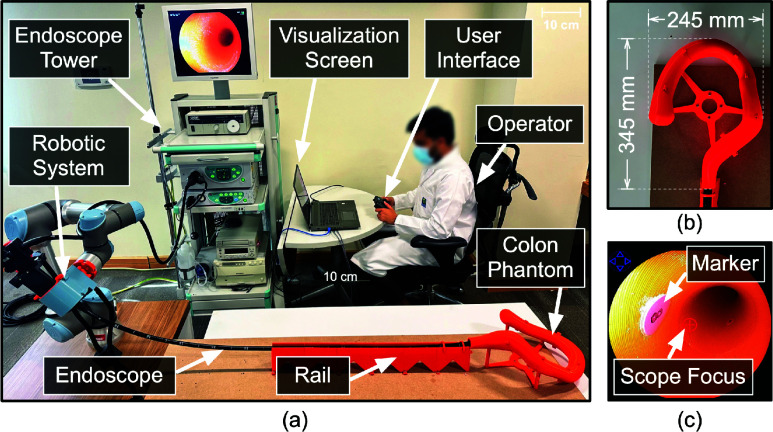
(a) Experimental setup used for Scenario-B. (b) 3D printed colon phantom. (c) View of the operative field acquired from the endoscope.

**FIGURE 6.**
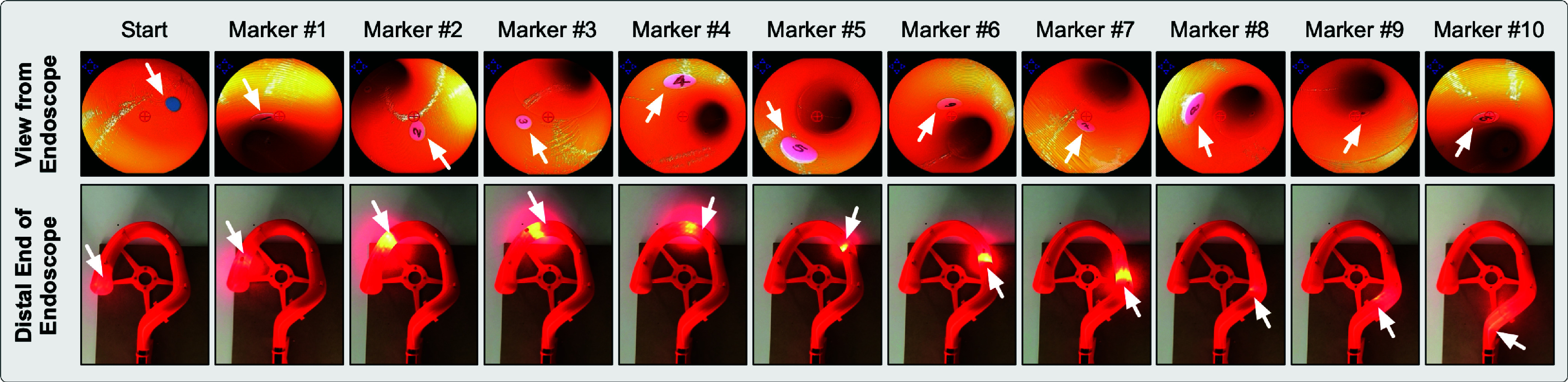
Placement of the circular markers representing polyps inside the colon phantom. The distance of the distal end of the endoscope from the orifice is mentioned below each marker.

Scenario-B simulated a polyp-targeting task during the withdrawal phase of a colonoscopy procedure [23]. All the movements of the endoscope were used (experimental setup shown in Figure 5a). A rail was used to prevent buckling and guide the endoscope to the orifice entrance of a 3D-printed colon phantom (Figure 5b). The task involved aligning circular markers representing polyps with the circled plus sign “ $\oplus $” rendered at the center of the operative field view representing endoscope’s focus (Figure 5c) [17]. Circular markers numbered 1 to 10, each of 8 mm diameter, were attached to the inner lining of the ascending, transverse, descending and sigmoid colon section of the phantom to be visualized by the colonoscope (Figure 6). During withdrawal phase, the subject could move to the next marker only if the previous marker was correctly visualized. This was validated by a moderator during the task. The parameters recorded for this scenario included: (a) the duration to traverse the markers (minutes), (b) the average time to align a target to the center of the screen once it appeared in the operating field (seconds), and (c) the number of clutch counts. Table 4 summarizes the scenarios used in the study.

**TABLE 4:** User Study

	Scenario A	Scenario B
Objective	Evaluate the effectiveness of user interfaces in accurately examining a specified diagnostic area.	Evaluating user interface effectiveness for navigating and visualizing targets inside tubular anatomical structures.
Setup	Operative field represented by an image with a tissue-like background.	3D-printed colon model featuring ascending, transverse, descending, and sigmoid sections.
Endoscope Movements	Movements limited to distal tip manipulation only.	Movements included translation, rotation, and distal tip manipulation.
Task	Navigate segmented track while keeping endoscope’s focus within boundaries (visually monitored).	Align markers representing polyps inside colon (moderator confirms alignment before proceeding).
Parameters Recorded	(a) Time to traverse the designated track, (b) Endoscope’s focus shifts outside the track, (c) Clutch counts	(a) Duration to traverse and align markers, (b) Average alignment time per marker after appearance on screen, (c) Clutch counts.

After completing the study, the subjects completed a NASA-TLX questionnaire to subjectively evaluate all the user interfaces and interaction modes. The questionnaire assessed mental demand, physical demand, temporal demand, performance, efforts, and frustration. A lower score reflected a better evaluation of the input method.

### D. Data Analysis

The order of user interfaces was randomized for each subject (simple randomization [29]) to eliminate bias, learning effect and subject-to-subject variation in the collected data. The recorded video streams were anonymized to conceal the subjects’ identities, as well as the interfaces and interaction modes used, before extracting the required parameters to prevent any confirmation bias. The videos were manually processed by a researcher to acquire the required parameters for statistical analysis. One-way ANOVA test was utilized to find whether there are significant mean differences across the 10 methods (combination of interfaces and interaction modes) and if it exists, Tukey’s Honest significant differences was used to identify the pairwise significance between the methods. A detailed model adequacy checking was performed to validate the data for normality assumption and equality of variances (homogeneity) prior to performing the one-way ANOVA. In case of assumption violations, Box-Cox algorithm recommendations were used to transform the variable such that the assumptions are no longer violated.

## IV. Results

**FIGURE 7.**
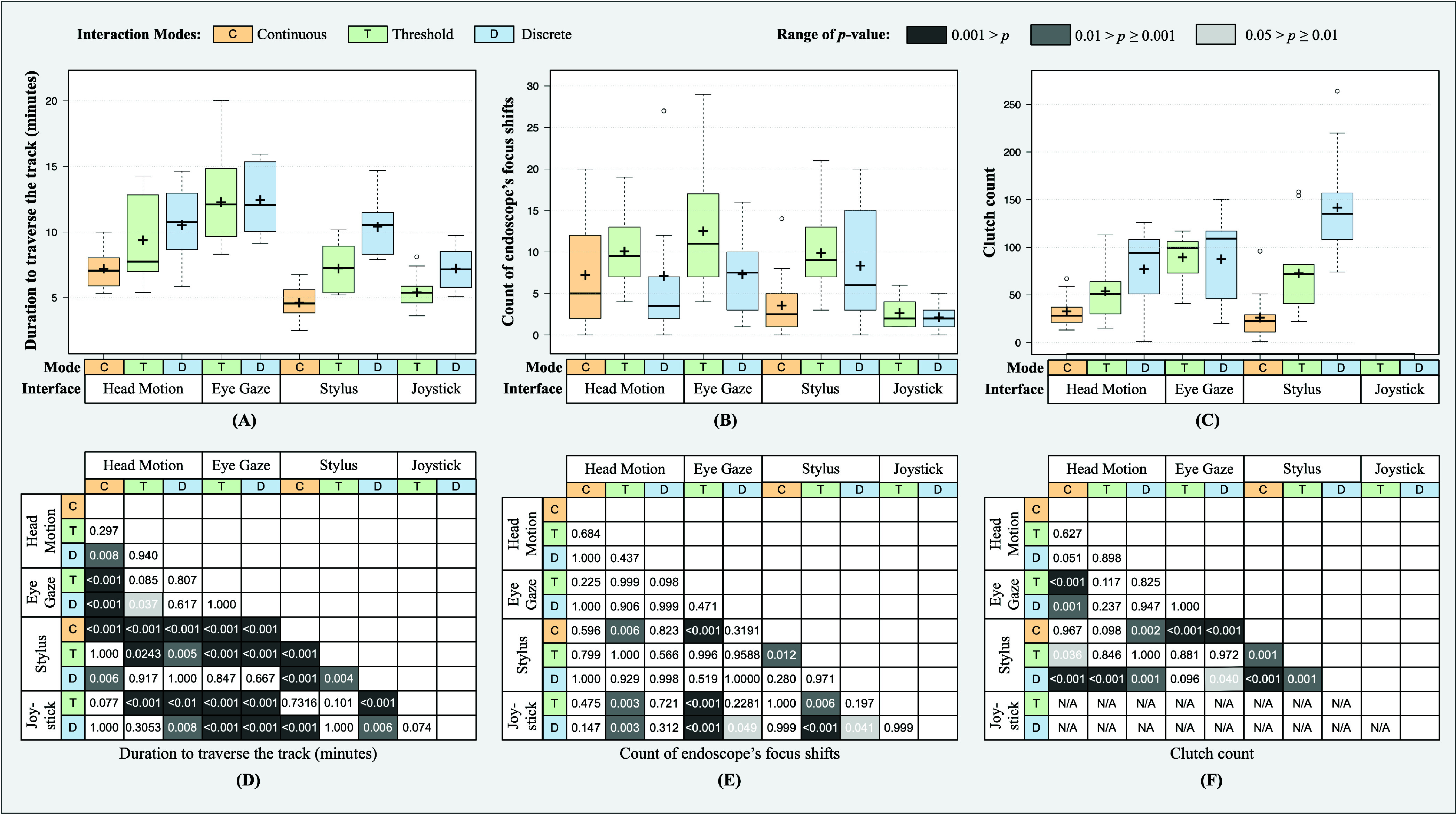
Box plots of the recorded parameters (Panels A, B, and C) in Scenario-A for four interfaces working under three interaction modes. The heat map (Panel D, E, and F) illustrates the pairwise significance of the corresponding recorded parameters among the user interfaces.

In this section, the notation *UserInterface* ${}_{\textit {(C,T,D)}}$ is used to denote an user interface operating under continuous (C), threshold (T), or discrete (D) interaction mode. Figure 7 presents the parameters recorded for Scenario-A. The duration to traverse the track using joystick and stylus was comparatively less than that required by other user interfaces. *Joystick* ${}_{\textit {(D,T)}}$ required less duration to traverse the track as compared to *head_motion* ${}_{\textit {(D)}}$, *eye_gaze* ${}_{\textit {(T,D)}}$ and *stylus* ${}_{\textit {(D)}}$. *Stylus* ${}_{\textit {(C)}}$ took less duration than *head_motion* ${}_{\textit {(C,T,D)}}$, *eye_gaze* ${}_{\textit {(T,D)}}$, *stylus* ${}_{\textit {(T,D)}}$ and *joystick* ${}_{\textit {(D)}}$. Similarly, *stylus* ${}_{\textit {(T)}}$ took less duration than *head_motion* ${}_{\textit {(D)}}$, *eye_gaze* ${}_{\textit {(T,D)}}$ and *stylus* ${}_{\textit {(D)}}$. *Head_motion* ${}_{\textit {(C)}}$ took less duration than *head_motion* ${}_{\textit {(D)}}$, *eye_gaze* ${}_{\textit {(T,D)}}$ and *stylus* ${}_{\textit {(D)}}$. In addition, *head_motion* ${}_{\textit {(T)}}$ took less duration than *eye_gaze* ${}_{\textit {(D)}}$ and *joystick* ${}_{\textit {(T)}}$. Both modes of joystick *Joystick* ${}_{\textit {(D,T)}}$ exhibited fewer counts of endoscope’s focus shifts as compared to *head_motion* ${}_{\textit {(T)}}$, *eye_gaze* ${}_{\textit {(T)}}$ and *stylus* ${}_{\textit {(T)}}$. In addition to this, *joystick* ${}_{\textit {(D)}}$ also had fewer counts of endoscope’s focus shifts as compared to *eye_gaze* ${}_{\textit {(D)}}$ and *stylus* ${}_{\textit {(D)}}$. It was also observed that *stylus* ${}_{\textit {(C)}}$ exhibited significantly lower counts of endoscope’s focus shifts as compared to *head_motion* ${}_{\textit {(T)}}$, *eye_gaze* ${}_{\textit {(T)}}$ and *stylus* ${}_{\textit {(T)}}$. The number of clutches used in *stylus* ${}_{\textit {(D)}}$ was significantly higher as compared to *head_motion* ${}_{\textit {(C,T,D)}}$, *eye_gaze* ${}_{\textit {(D)}}$ and *stylus* ${}_{\textit {(C,T)}}$. Alternatively, the continuous mode of stylus took a significantly smaller number of clutch counts as compared to *head_motion* ${}_{\textit {(D)}}$, *eye_gaze* ${}_{\textit {(T,D)}}$ and *stylus* ${}_{\textit {(T)}}$. Similarly, *head_motion* ${}_{\textit {(C)}}$ used fewer clutches as compared to *eye_gaze* ${}_{\textit {(T,D)}}$ and *stylus* ${}_{\textit {(T)}}$.

**FIGURE 8.**
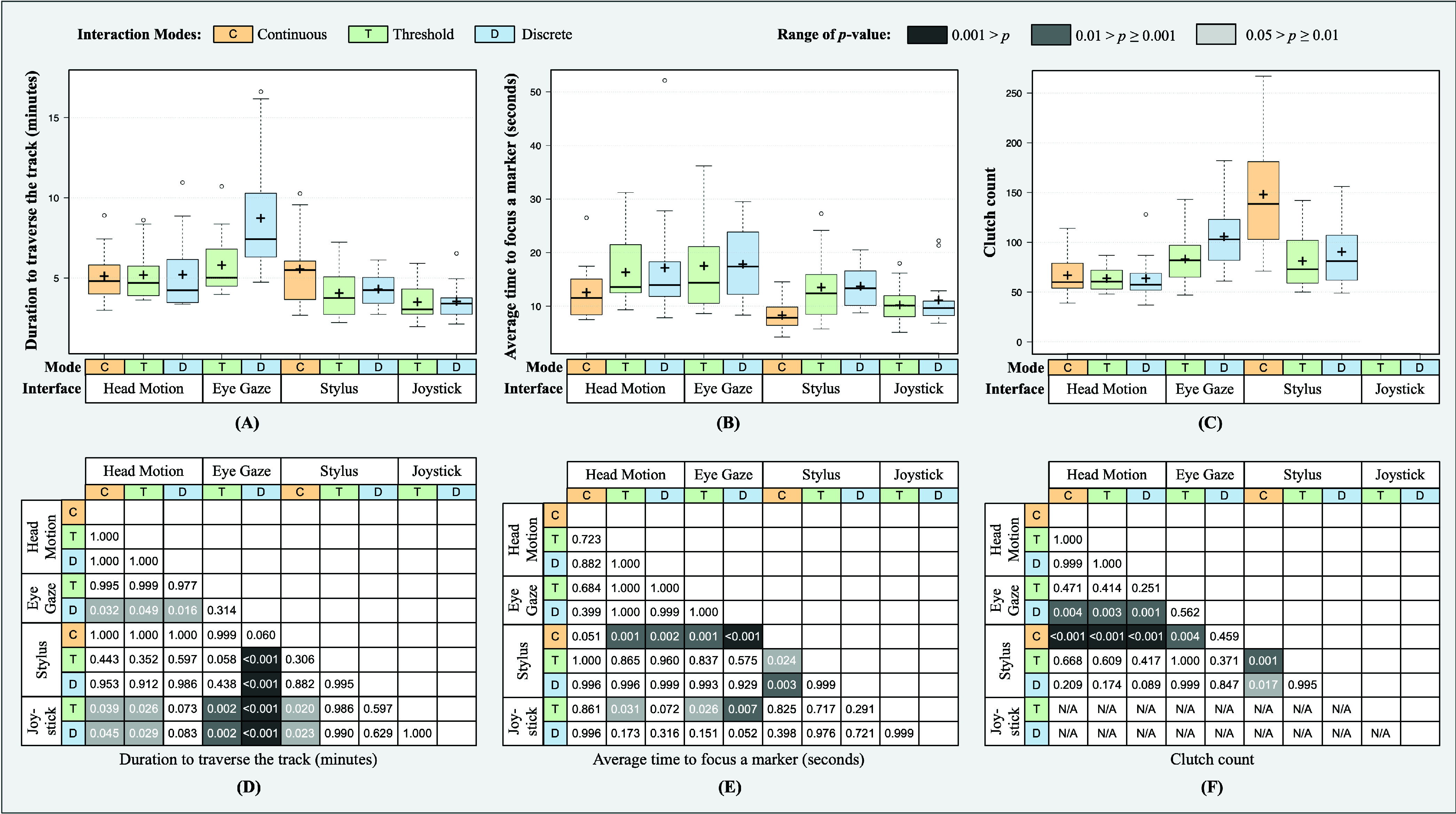
Box plots of the recorded parameters (Panels A, B, and C) in Scenario-B for four interfaces working under three interaction modes. The heat map (Panel D, E, and F) illustrates the pairwise significance of the corresponding recorded parameters among the user interfaces.

The parameters recorded for Scenario-B are presented in figure 8. The duration to traverse the track using *joystick* ${}_{\textit {(T,D)}}$ was lower in comparison to *head_motion* ${}_{\textit {(C,T)}}$, *eye_gaze* ${}_{\textit {(T,D)}}$ and *stylus* ${}_{\textit {(C)}}$. It was also found that the duration to traverse the track in case of *eye_gaze* ${}_{\textit {(D)}}$ was significantly higher than *head_motion* ${}_{\textit {(C,T,D)}}$ and *stylus* ${}_{\textit {(T,D)}}$. The average time to focus a marker in *stylus* ${}_{\textit {(C)}}$ was significantly lower than *head_motion* ${}_{\textit {(C,T,D)}}$, *eye_gaze* ${}_{\textit {(T,D)}}$ and *stylus* ${}_{\textit {(T,D)}}$. Similarly, *joystick* ${}_{\textit {(T)}}$ took less average time to focus a marker than *head_motion* ${}_{\textit {(T)}}$ and *eye_gaze* ${}_{\textit {(T,D)}}$. Additionally, *joystick* ${}_{\textit {(D)}}$ was also found to have significantly less average time to focus a marker in comparison to *eye_gaze* ${}_{\textit {(D)}}$. The clutch count used in *stylus* ${}_{\textit {(C)}}$ was significantly higher than *head_motion* ${}_{\textit {(C,T,D)}}$, *eye_gaze* ${}_{\textit {(T)}}$ and *stylus* ${}_{\textit {(T, D)}}$. Similarly, the clutch count in *eye_gaze* ${}_{\textit {(D)}}$ was significantly higher than *head_motion* ${}_{\textit {(C,T,D)}}$. The scores of NASA-TLX questionnaire are presented in form of box-plots in the supporting document.

## V. Discussion

The NASA-TLX data collected was analyzed and used to draw insights related to user experience. Under Scenario A, the following observations were made for the recorded parameters:

(a) *Duration to traverse the track:* The task duration reflected the overall performance of the interfaces and the interaction modes. Joystick took the least amount of time to complete the task in comparison to threshold and discrete modes of other interfaces. The joystick performed well due to its ease of operation, intuitive controls, and absence of clutch. The continuous interaction modes of head-motion and stylus device also took less duration, as they allowed for variable speed control, facilitating precise endoscope movement and faster traversal along the curved section of the track. It took significantly longer in case of eye-gaze (for both discrete and threshold modes), head-motion (discrete mode), and stylus device (discrete mode). The discrete modes of each user interface took longer than their respective counterparts.
(b) *Count of endoscope’s focus shifts:* Lesser the number of focus shifts, more optimal is the track traced by the subject. Joystick exhibited the least number of focus shifts as compared to the threshold and discrete modes of other interfaces. This was followed by the continuous interaction mode of stylus device. The subjects were able to prevent focus shift out of the track, but in cases it did, the additional distance moved out of the track was negligible compared to other interfaces.
(c) *Clutch count*: The number of clutches used by the subjects reflected the ability to control the endoscope’s movements without frequent adjustments. The continuous modes of head motion and stylus device exhibited the least number of clutches required by the subject to traverse through the track. This is due to the ability to precisely control the movements using variable speed, i.e., slow around the curves and faster across straight lines, and keeping the head/stylus still to halt the movement. The discrete mode of stylus device exhibited the highest number of clutch counts since the subject had to start and stop the movement several times. This is due to the inability to move the endoscope in a continuous path. The subjects adapted a ladder approach to trace the track. To move in a ladder fashion, the subject frequently started and stopped the motion either by bringing the stylus back to the start position or by using the clutch. It was easy and preferred by the subjects to use the clutch, leading to an increase in the clutch count. In the case of discrete interaction modes of head motion and eye gaze, the subject had the ability to return to the neutral head/eye position naturally. This did not exist for the stylus device and as a result the subjects preferred engaging the clutch for movements.

Under Scenario B, the following observations were made for the recorded parameters:

(a) *Duration to traverse the colon:* The usage of joystick (in both the interaction modes) to traverse the colon, neither suffered from limited range of motion nor were the subjects overwhelmed with clutch to trigger the movement, hence, the total duration to complete the task was minimal. Although continuous interaction mode is expected to traverse faster due to variable speed, the limited range within which the operator’s head, eye-gaze, and the stylus could be moved hindered faster completion of the task. Eye-gaze under discrete interaction mode took the longest duration. The subjects struggled to stabilize their eye-gaze when moving their heads backward to retract the endoscope. This caused inaccurate movements leading to repeated attempts to direct the endoscope correctly.
(b) *Average time to focus a marker:* The average time to focus on a target signified movements similar to Scenario-A. Once the marker was visualized, the subject manipulated the distal end of the endoscope using up/down and left/right movements and without relying on the retraction movement. Due to this, the results were in line with Scenario-A. The continuous mode of the stylus device required significantly less average time to reach target as compared to head-motion, eye-gaze and other modes of stylus device.
(c) *Clutch count:* The clutch count was predominantly determined by the insertion/retraction movements. Stylus device operating under continuous interaction mode required the highest number of clutch counts. As the stylus device has a limited range of motion, it required the subjects to repeat the same motion multiple times for retraction leading to an increase in the clutch count.

Although head-motion and eye-gaze user interfaces scored lower on the recorded parameters, they provide hands-free control of the endoscope through the actuation system. This is beneficial particularly during therapeutic interventions that require manual control of instruments inserted through the endoscope. The actuation system equipped with head-motion and eye-gaze can minimize errors caused due to miscommunications between the endoscopist and the assistant handling the instruments [30]. Additionally, the head motion based user interface can be extended to support instrument control [15].

The user study demonstrated the use of continuous interaction mode for head-motion and stylus. The interaction mode is novel as compared to the existing control techniques used in manipulation of flexible endoscopes. Previous works were limited to either threshold, discrete, or kinematics based stepwise movement of the endoscope’s distal end. Systems like EndoDrive offered preset speed levels, requiring the speed to be set before movement, whereas the proposed continuous interaction mode enabled movement with real-time adjustment of speed. The improved performance using continuous interaction mode is demonstrated by its ability to focus on a selected target while keeping the endoscope’s shaft stationary (Scenario-A) as well as targeting structures during the retraction of the endoscope (Scenario-B).

The joystick interface outperformed the stylus. This is consistent with previous findings [23]. In scenario B, the extended task completion time with the stylus was attributed to the limited range of motion caused by spring force effect [19]. Implementing an alternative interface, such as a foot pedal, for endoscope insertion and retraction could potentially enhance performance when using the stylus.

To establish a baseline, additional tests (n = 11) using manual endoscope control were conducted and two key limitations of this method were observed. First, this method relied on two rotating knobs operated by the left-hand thumb of the user, making simultaneous and precise control difficult. Second, in Scenario-A, achieving the required precision to follow the track smoothly was nearly impossible, resulting in unstable trajectories. In Scenario-B, manual control led to an average task time of $3~\pm ~0.5$ minutes, comparable to some tested interfaces, but with reduced user comfort. These findings emphasize the limitations of manual operation and the advantages of the proposed actuation system and interfaces.

While the study was designed to ensure minimal bias and avoid learning effects, the impact of the fixed scenario setup and potential learning curves across different interfaces is acknowledged. The fixed scenario setup throughout the study, which could have allowed participants to perform better in later trials, was addressed by randomizing the order of interface–interaction mode combinations for each participant. To support a more accurate assessment, evaluation metrics, such as the average time to focus on a marker (in scenario B), ensured that the results reflected not only the time taken for target identification, but also the ease of alignment, which was purely dependent on the interface–interaction mode. The learning effects were controlled by limiting each interface–interaction mode to a single trial per participant. The intuitiveness and ease of use of each interface may have influenced adaptation speed differently. The joystick and stylus interfaces, being more direct and familiar, likely resulted in shorter learning curves, while the eye tracker may have required more time to adjust. Similarly, within the interaction modes, the continuous mode of the head tracker and stylus may have enabled shorter learning curves due to their direct mapping with the input.

The study has two limitations. First, the subjects did not include expert endoscopists. Since the user study was conducted with an endoscope actuation system and a user interface, the manual hand-eye coordination skills required for traditional endoscope manipulation did not impact the study’s outcomes. Second, an inanimate 3D-printed colon model was used. While similar models have been employed in previous studies [31], they do not accurately simulate tissue properties. Despite this limitation, using the model was essential for maintaining a consistent environment across different subjects. Integrating a force-sensing mechanism to detect interactions between the endoscope shaft and the colon wall could improve navigation with user interfaces, potentially reducing colon wall stretching and minimizing the risk of perforation [32]. Future studies involving clinical end users, such as surgeons and endoscopists, will be essential for advancing the system to higher Technology Readiness Levels (TRL). Feedback from clinicians will support refinement of the system design and user interfaces to better align with clinical requirements and facilitate progression toward TRL 7 and beyond.

## VI. Conclusion

The study provided insights into the use of various user interfaces for actuated maneuvering of endoscopes during flexible endoscopy. The joystick consistently outperformed other interfaces across all interaction modes. Performance among the other interfaces varied based on different parameters and scenarios. Head-motion and eye-based interfaces enabled hands-free manipulation of the endoscope. The continuous interaction modes of the head-motion and stylus interfaces facilitated faster traversal of a localized diagnostic area using the distal end of the endoscope without the need for insertion or retraction. This study will aid in making informed decisions when selecting a suitable user interface and interaction mode for flexible endoscopy applications, as well as in enhancing the performance of actuation systems.
